# Restriction of Aerobic Metabolism by Acquired or Innate Arylsulfatase B Deficiency: A New Approach to the Warburg Effect

**DOI:** 10.1038/srep32885

**Published:** 2016-09-08

**Authors:** Sumit Bhattacharyya, Leo Feferman, Joanne K. Tobacman

**Affiliations:** 1 Department of Medicine, University of Illinois at Chicago, Chicago, IL 60612, USA; 2 Jesse Brown VA Medical Center, Chicago, IL 60612 USA

## Abstract

Aerobic respiration is required for optimal efficiency of metabolism in mammalian cells. Under circumstances when oxygen utilization is impaired, cells survive by anerobic metabolism. The malignant cell has cultivated the use of anerobic metabolism in an aerobic environment, the Warburg effect, but the explanation for this preference is not clear. This paper presents evidence that deficiency of the enzyme arylsulfatase B (ARSB; N-acetylgalactosamine 4-sulfatase), either innate or acquired, helps to explain the Warburg phenomenon. ARSB is the enzyme that removes 4-sulfate groups from the non-reducing end of chondroitin 4-sulfate and dermatan sulfate. Previous reports indicated reduced ARSB activity in malignancy and replication of the effects of hypoxia by decline in ARSB. Hypoxia reduced ARSB activity, since molecular oxygen is needed for post-translational modification of ARSB. In this report, studies were performed in human HepG2 cells and in hepatocytes from ARSB-deficient and normal C57BL/6J control mice. Decline of ARSB, in the presence of oxygen, profoundly reduced the oxygen consumption rate and increased the extracellular acidification rate, indicating preference for aerobic glycolysis. Specific study findings indicate that decline in ARSB activity enhanced aerobic glycolysis and impaired normal redox processes, consistent with a critical role of ARSB and sulfate reduction in mammalian metabolism.

The malignant cell has cultivated the use of anerobic metabolism in an aerobic environment, the Warburg effect, but the explanation for this preference is not clear^1^,^2^. This paper presents evidence that deficiency of the enzyme arylsulfatase B (ARSB; N-acetylgalactosamine 4-sulfatase), either innate or acquired, helps to explain the Warburg phenomenon. ARSB removes the 4-sulfate group from the non-reducing end of the sulfated glycosaminoglycans, chondroitin 4-sulfate (C4S) and dermatan sulfate (DS) and regulates their degradation^3^,^4^. C4S and DS are fundamental constituents of mammalian cells and the extracellular matrix. C4S is composed of alternating residues of N-acetyl-D-galactosamine 4-sulfate and D-glucuronate, with β-1,3 and β-1,4 glycosidic links. Dermatan sulfate has iduronate residues instead of glucuronate. C4S is a component of several proteoglycans, including the lecticans versican, neurocan, and aggrecan. C4S bears a negative charge due to its abundant sulfate groups. Interactions of C4S with cations or with positively charged proteins establish the structural and functional properties of chondroitin sulfate and other sulfated GAGs, including heparan sulfate and keratan sulfate. Since the only known function of ARSB is to remove the terminal sulfate group at the non-reducing end of N-acetylgalactosamine 4-sulfate, the effect of ARSB on metabolism must be due to impact on C4S or DS structure or function, or on the availability of sulfate. Specific chondroitin sulfotransferases act to produce chondroitin sulfates, including C4S, chondroitin 6-sulfate, chondroitin 4,6,-disulfate, and chondroitin 2,6-disulfate. The sulfotransferases require 3′-phosphoadenosine 5′-phosphosulfate (PAPS) as sulfate donor. PAPS is made by PAPS synthetase using ATP and inorganic sulfate^5^,^6^,^7^.

Increased C4S sulfation following decline in ARSB was shown to lead to transcriptional events, mediated either by galectin-3 or by the non-receptor tyrosine phosphatase SHP2 8,9,10,11. Galectin-3 binding to C4S was less when ARSB activity was less. Galectin-3 then interacted with transcription factors AP-1 and Sp1, leading to promoter activation and increased expression of versican, hypoxia inducible factor-1, and Wnt 9A, as reported^8^,^9^,^10^. In contrast, SHP2 bound more tightly to the more highly sulfated C4S present when ARSB activity was less. This enhanced binding led to reduced phosphatase activity, sustained phosphorylation of critical tyrosine residues, and prolonged activation of associated MAPK signaling pathways^11^.

ARSB appears to be a tumor suppressor in human tissues, since decline in ARSB was associated with human prostate, mammary, and colonic malignancies^12^,^13^,^14^,^15^,^16^. Lower ARSB was associated with more advanced prostate and colonic malignancies. In human epithelial cells, decline in ARSB replicated the effects of hypoxia^9^. Both decline in ARSB and hypoxia had similar effects on the expression of 84 genes in a hypoxia gene array^9^. Also, both hypoxia and silencing of ARSB increased the abundance of total sulfated glycosaminoglycans and chondroitin 4-sulfate (C4S). Hypoxia reduced the ARSB activity in cultured human bronchial and colonic epithelial cells^9^. This effect was attributed to a requirement for oxygen for ARSB function, since molecular oxygen is needed for the post-translational modification of ARSB^17^,^18^.

Congenital deficiency of ARSB leads to Mucopolysaccharidosis (MPS) VI, a lysosomal storage disease, characterized by the accumulation of C4S and DS throughout the body. MPS VI is manifested by growth retardation, skeletal deformities, organ failure, valvular heart disease, and shortened lifespan. ARSB was previously regarded as only a lysosomal enzyme, but has been shown to be present and functional in the cell membrane of epithelial and endothelial cells^12^,^13^,^14^,^19^,^20^.

The experiments presented in this report were performed in hepatic tissue and hepatocytes from ARSB-null and C57BL/6J control mice and in human HepG2 cells. The studies indicate significant metabolic defects in mammalian cells when ARSB is decreased. Effects include: decreased mitochondrial membrane potential; enhanced extracellular acidification; reduced oxygen consumption; decline in NADH and NADPH oxidase activity; and decline in NAD+/NADH and NADP+/NADPH ratios. These effects are consistent with enhanced aerobic glycolysis (the Warburg effect) when ARSB activity is less.

In plants, bacteria, and protists, sulfate reduction has been elucidated as a type of anerobic metabolism in which sulfate acts as an electron acceptor^21^,^22^,^23^,^24^,^25^,^26^,^27^,^28^,^29^,^30^. In plants, assimilatory sulfate reduction is an energy-requiring process which involves the activation of sulfate to adenosine 5′-phosphosulfate (APS)^21^. Sulfate in APS is reduced to sulfite, then further reduced to sulfide, and then incorporated into cysteine or other organo-sulfur compounds. Alternatively, APS is phosphorylated to 3′-phosphoadenosine 5-′phosphosulfate (PAPS), and sulfate is transferred to other molecules by sulfotransferase reactions^22^,^23^,^24^,^25^,^26^,^27^. Decline in sulfate uptake in plants has been associated with mitochondrial abnormalities and changes in redox status^28^.

An alternative, energy-producing process of dissimilatory sulfate reduction has also been described^28^,^29^,^30^. In sulfate-reducing bacteria and archaea, dissimilatory sulfate reduction, leads to production of inorganic sulfide or hydrogen sulfide. The reduction of sulfate directly to sulfite has been reported as energetically unfavorable in microorganisms. Hence, conversion of sulfate into APS by ATP sulfurylase is a necessary step in sulfate reduction. The enzymes adenyl sulfate reductase, which utilizes ferredoxin and NADH, and dissimilatory sulfite reductase act to enable the production of sulfide^28^,^29^,^30^.

The studies in this report suggest a critical role for ARSB in mammalian sulfate reduction. The decline of ARSB in malignancy suggests a link between malignancy and impairment of sulfate reduction, due to reduced availability of sulfate when ARSB activity is diminished. The findings that follow indicate profound effects of decline in ARSB on oxidative metabolism in mammalian cells, consistent with the Warburg effect.

## Results

**Figure 1:**
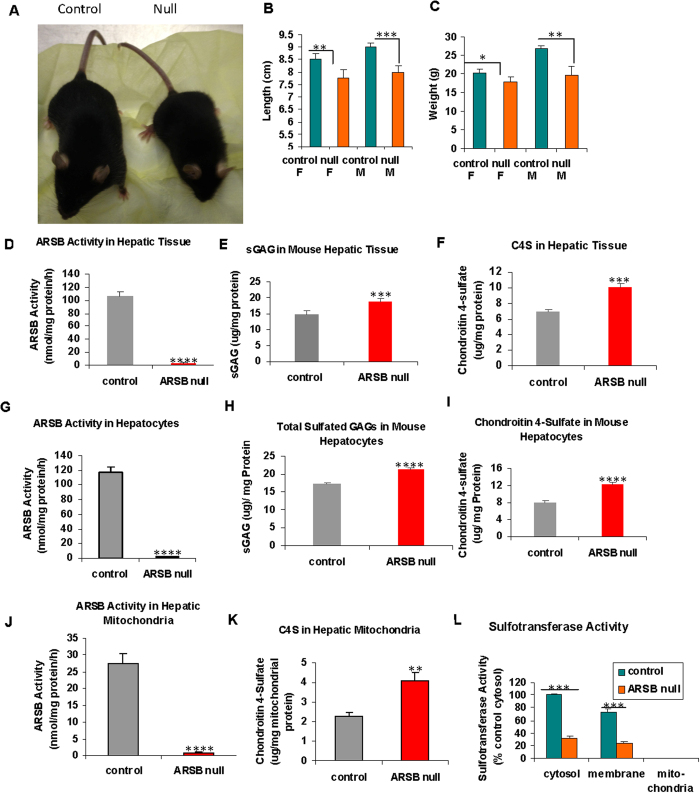
Size of ARSB-null mice, and measurements of arylsulfatase B activity, chondroitin 4-sulfate, and sulfotransferase activity. (**A**) Images of the ARSB control and null mice show a significant size disparity. (**B**) At twelve weeks, ARSB-null mice were significantly smaller than control mice (total n = 17). (**C**) At twelve weeks, the ARSB-null mice weighed significantly less than the control mice. (**D**) ARSB activity was significantly less in the ARSB-null mouse hepatic tissue than in the age-matched normal C57BL/6J controls. Values were similar for male and female mice. (**E**) Consistent with the known reduction in ARSB activity in the hepatic tissue of ARSB-null mice, the total sulfated glycosaminoglycan (GAG) content in the hepatic tissue of the ARSB-null mice was significantly greater than in the controls (n = 20). (**F**) The chondroitin 4-sulfate (C4S) content was also significantly greater in the ARSB-null mice. The increase in total sulfated GAG was largely attributable to the increase in C4S. (**G**) In primary hepatocytes from the ARSB null and C57BL/6J control mice, the ARSB activity in the hepatocytes from the ARSB-null mice was significantly less than from the controls (pn = 6). (**H**) Consistent with the decline in ARSB activity, the total sulfated GAG was markedly increased in the primary hepatocytes from the ARSB-null mice, compared to the normal control (n = 6). (**I**) Similarly, the C4S in the primary hepatocytes from the ARSB-null mice was significantly greater than in the controls. (**J**) The mitochondria isolated from the hepatic tissue of the control mice had lower ARSB activity than the primary hepatocytes from the control hepatic tissue (27.6 ± 2.8 nmol/mg protein/h vs. 117.8 ± 6.6 nmol/mg protein/h). In the ARSB null mice, the mitochondrial ARSB activity was virtually absent. (**K**). The chondroitin 4-sulfate level in the hepatic mitochondria was higher in the ARSB-null mice than in the control mice, but was markedly less than in the primary hepatocytes. (**L**) Sulfotransferase activity was absent in the mitochondrial preparation from both control and ARSB-null mouse hepatic tissue (n = 3). [ARSB = arylsulfatase B (N-acetylgalactosamine 4-sulfatase); C4S = chondroitin 4-sulfate; c = control; F = female; M = male; null = ARSB-null; ST = sulfotransferase; WT = weight]

Like patients with MPS VI, ARSB- null mice are small, with lower body weight and length than age- and gender-matched C57BL/6J controls (total n = 17) (Fig. 1A). Body length (Fig. 1B) and body weight (Fig. 1C) of ARSB-null male and female mice were significantly less than in matched, control mice at ~12 weeks.

ARSB activity in the hepatic tissue of the null mice was less than 2% of the value in the control mice (2.2 ± 0.8 nmol/mg protein/h vs. 107.5 ± 5.5 nmol/mg protein/h) (Fig. 1D). Consistent with this decline in ARSB activity, the total sulfated glycosaminoglcyans (GAGs) (Fig. 1E) and chondroitin 4-sulfate (C4S) (Fig. lF) in the hepatic tissue of the ARSB-null mice were significantly greater (p < 0.001) than in the control mice. ARSB activity in primary hepatocytes from the control mice and the ARSB-null mice (Fig. 1G) was similar to the values in the mouse hepatic tissue. Consistent with this result, the total sulfated GAGs (Fig. 1H) and C4S (Fig. 1I) levels in the primary hepatocytes were also similar to those in the hepatic tissue. In contrast, ARSB activity was much lower in the isolated mitochondria from the control hepatic tissue (Fig. 1J) than in the primary hepatocytes from the control hepatic tissue (27.6 ± 2.8 nmol/mg protein/h vs. 117.8 ± 6.6 nmol/mg protein/h). The C4S levels were higher in the mitochondria of the ARSB-null mice than in mitochondria of the normal control mice (4.11 ± 0.41 μg/mg mitochondrial protein vs. 2.28 ± 0.10 μg/mg mitochondrial protein) (Fig. 1K). Values of ARSB were negligible in the hepatic tissue, primary hepatocytes, and mitochondria of the ARSB-null mice.

ARSB was effectively silenced by siRNA in the HepG2 cells, as previously reported^11^. In the HepG2 cells, siRNA treatment reduced the ARSB activity to ~13 nmol/mg protein/h from a baseline value of ~99 nmol/mg protein/h at 24 h^11^.

Interestingly, sulfotransferase activity was absent in the mitochondrial-containing fraction of the hepatic tissue from both the ARSB-null and control mice (Fig. 1L). This finding suggests that sulfate was not utilized for sulfotransferase reactions in the mitochondria of the normal mouse.

Determinations of enzyme activity (Table 1) demonstrate the successful separation of mitochondria, peroxisomes, and lysosomes by the organelle preparation procedure. Succinate dehydrogenase was highest in mitochondria, in contrast to highest activity of catalase in peroxisomes and acid phosphatase in lysosomes.

### Increase in extracellular acidification and decline in oxygen consumption rates

**Figure 2:**
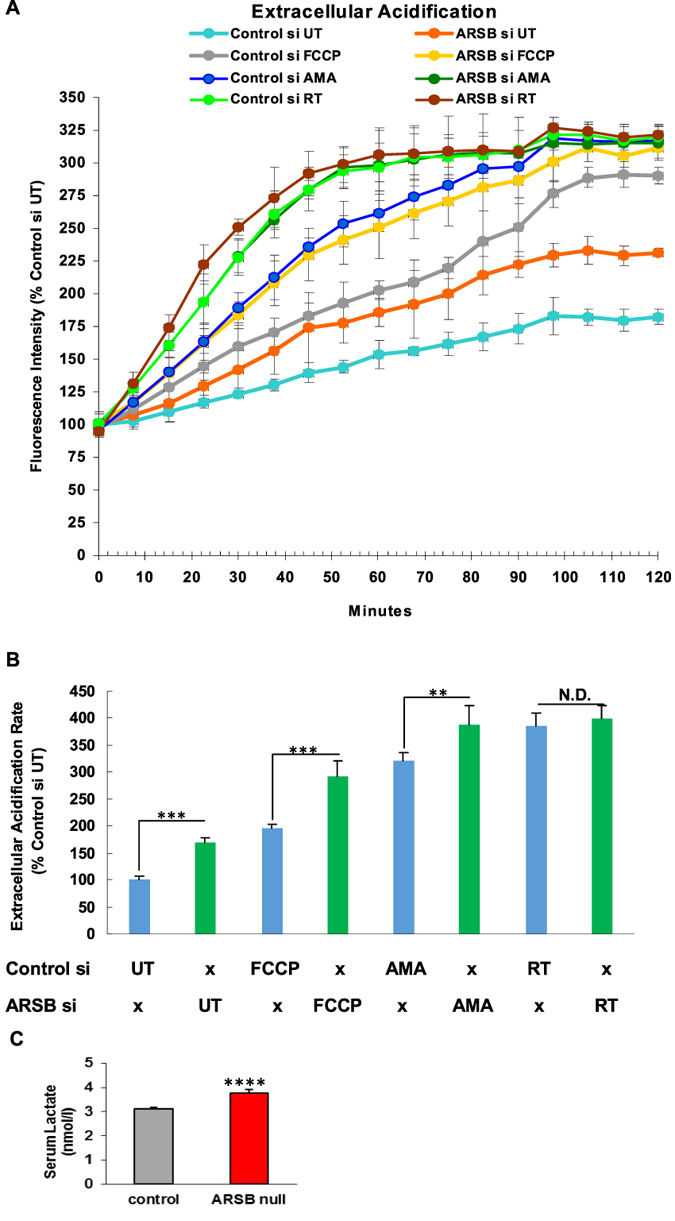
Increased extracellular acidification rate following ARSB-silencing in HepG2 cells, and increased serum lactate in ARSB-null mice. (**A**) Extracellular acidification rate (ECAR) was measured in HepG2 cells following ARSB-silencing and control silencing (n = 6 samples for each condition). Following ARSB silencing, the ECAR was increased at all time points relative to the control-silenced cells. Exposure to antimycin A, a complex III inhibitor, rotenone, a complex 1 inhibitor, and FCCP, an uncoupler of the electron transport chain, all increased the ECAR (n = 6). (**B**) The value of the ECAR was calculated as the slope from 0 to 60 minutes. Values were significantly different between control and ARSB-silenced untreated HepG2 cells and between control and ARSB-silenced AMA- and FCCP- treated cells, but not for the rotenone-treated cells. Also, ARSB-silencing with AMA approximates the steeper slope of the rotenone-treated HepG2 cells. These results raise the possibility that the predominant effect of ARSB silencing was on Complex 1 (n = 6). (**C**) Consistent with the marked increase in ECAR following ARSB silencing, serum lactate was significantly increased in the ARSB-null mice (n = 12).

Experiments were performed to further assess the impact of decline in ARSB and the associated decline in available sulfate on mitochondrial function and cellular metabolism. In ARSB-silenced HepG2 cells, the extracellular acidification rate (ECAR) was increased, compared to control-silenced cells (Fig. 2A). The slope of the ECAR n the first 60 minutes was determined, and was significantly greater following ARSB silencing (p < 0.001) (Fig. 2B). Increases in ECAR in the ARSB-silenced cells were sustained throughout 120 minutes of measurement.

Cells were treated at time 0 with either antimycin A (AMA), which acts predominantly on Complex 3, rotenone (RT), which acts predominantly on Complex 1, or carbonyl cyanide-p-trifluoromethoxyphenylhydrazone (FCCP), which acts as an uncoupling agent. All ECAR values increased, and the increases in the ARSB-silenced cells were consistently more than in the control cells. The differences between the rotenone-treated ARSB-silenced cells and the rotenone-treated control cells were less than for the AMA-treated or FCCP-treated cells, raising the possibility that ARSB-silencing acts most on Complex I.

Consistent with the increase in ECAR following ARSB silencing, serum lactate in the ARSB-null mice was significantly greater than in the control mice (Fig. 2C).

**Figure 3:**
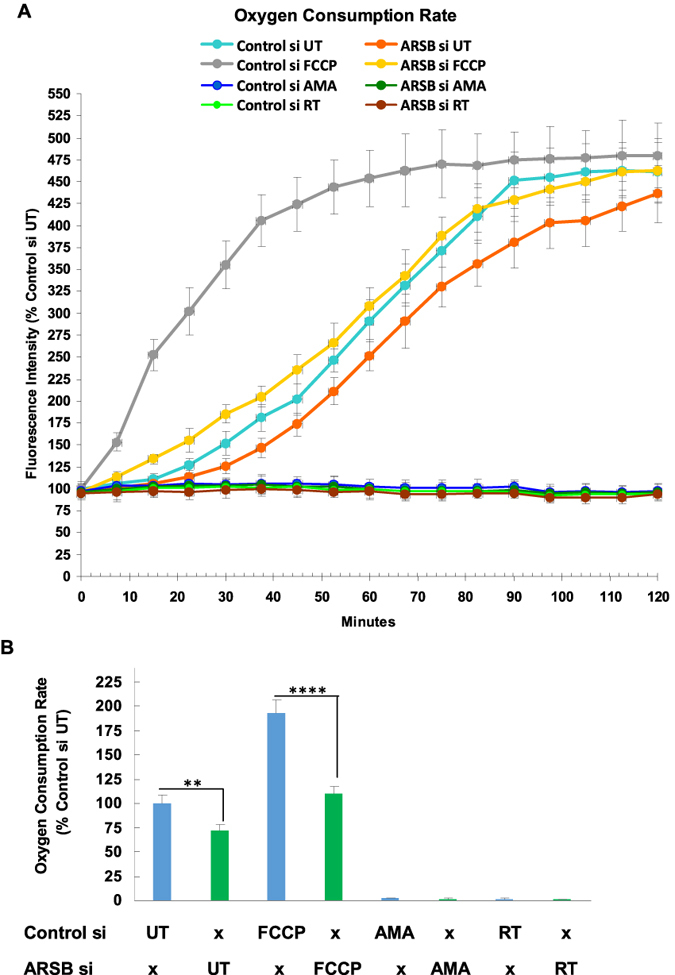
Decline in oxygen consumption rate following ARSB silencing in HepG2 cells. (**A**) The oxygen consumption rate (OCR) was greater for control-silenced than ARSB-silenced HepG2 cells at all time points. Values for rotenone- and AMA-treated cells were virtually undetectable at all time pints, with no effect of ARSB silencing discernible. In contrast, ARSB- silencing reduced the OCR at all time points compared to the untreated control. The ARSB-silenced with FCCP-treatment values were greater than the FCCP-alone values. This suggested that ARSB-silencing also had an impact on the electron transport chain and the proton gradient (n = 6). (**B**) The values for the slopes from 0 to 60 min were significantly different for the untreated ARSB-silenced HepG2 cells and the control-silenced cells, and for the FCCP-exposed ARSB-silenced and control-silenced cells. [AMA = antimycin A; ARSB = arylsulfatase B; ECAR = extracellular acidification rate; FCCP = carbonyl cyanide-p-trifluoromethoxyphenylhydrazone; N.D. = no difference; OCR = oxygen consumption rate; RT = rotenone; UT = untreated].

To test oxygen utilization, oxygen consumption rate was measured in the ARSB- and control-silenced HepG2 cells. In contrast to the increase in ECAR following ARSB silencing, the oxygen consumption rate (OCR) was consistently less at all time points (Fig. 3A). The slope of the OCR values for the first 60 minutes showed significant differences in the untreated cells and following addition at time 0 of FCCP in the ARSB-silenced HepG2 cells (Fig. 3B). Following treatment with either rotenone or antimycin A at time 0, oxygen consumption was completely inhibited in both the ARSB- and control- silenced cells. Following the addition of FCCP, which carries protons across the inner mitochondrial membrane and dissipates the electrochemical gradient, mitochondrial OCR was increased as expected, in an attempt to sustain the membrane potential.

### Reduction of ARSB inhibits mitochondrial membrane potential and Complex 1 activity

**Figure 4:**
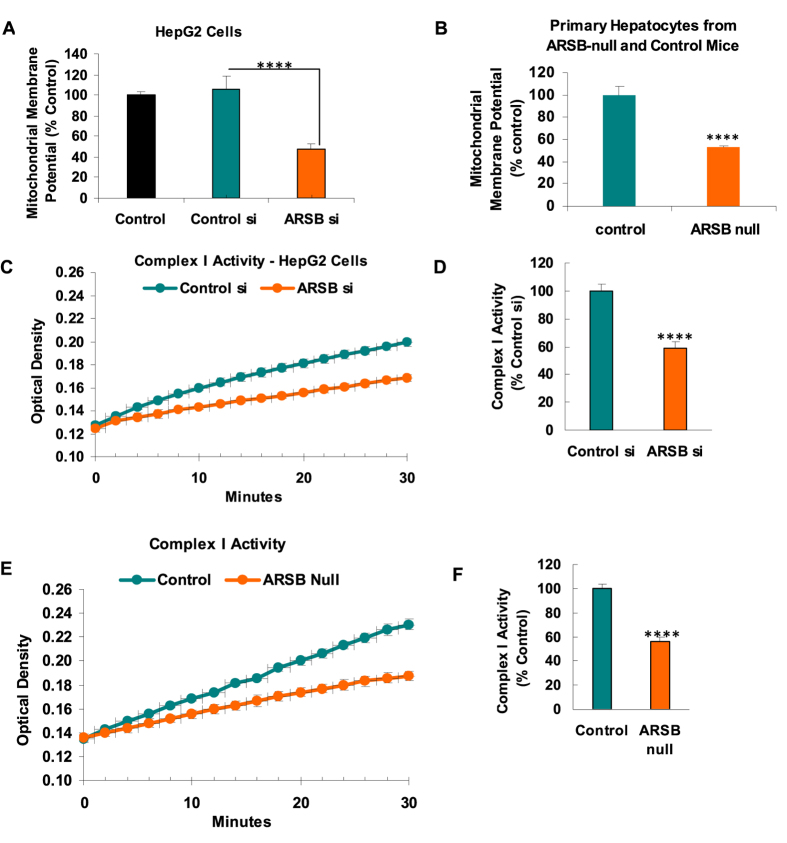
Mitochondrial membrane potential and Complex 1 activity in ARSB-null mice. (**A**) Mitochondrial membrane potential was measured in the HepG2 cells and shown to be significantly less when ARSB was silenced (n = 6; one-way ANOVA with Tukey-Kramer post-test). (**B**) Similarly, the mitochondrial membrane potential in the mitochondria from the ARSB-null primary hepatocytes was significantly less than in hepatocytes from the C57BL/6J control mice (n = 6). (**C**) The activity of Complex 1 was determined by an NADH dehydrogenase activity assay which showed marked reduction of activity in the mitochondria of the HepG2 cells following ARSB silencing, compared to control (n = 6). (**D**) Graphical representation of the slope of the activity shows the significant difference following ARSB silencing. (**E**) Similarly, Complex 1 activity was markedly reduced in the crude mitochondrial fraction from ARSB-null mouse hepatic tissue, compared to the value in the C57BL/6J (n = 6). (**F**) Graphical representation of the decline in Complex 1 activity of the ARSB-null mouse hepatic tissue, compared to the control [ARSB = arylsulfatase B; ER = endoplasmic reticulum; MMP = mitochondrial membrane potential].

To assess the impact of decline in ARSB on the mitochondrial membrane, mitochondrial membrane potential (MMP) was measured by the J10 dye, in ARSB-silenced and control-silenced HepG2 cells (Fig. 4A) and in primary hepatocytes from ARSB-null and control C57BL/6J mice (Fig. 4B). MMP was significantly less following ARSB siRNA, as compared to control values (p < 0.0001).

To further address the effects of decline in ARSB on mitochondrial function, the NADH dehydrogenase activity of Complex 1 was measured in HepG2 cells following ARSB silencing (Fig. 4C,D) and in the mitochondria from hepatic tissue of ARSB-null and control mice (Fig. 4E,F). Complex 1 activity was markedly reduced when ARSB was less (p < 0.0001), consistent with the observed declines in mitochondrial membrane potential and oxygen consumption.

**Figure 5:**
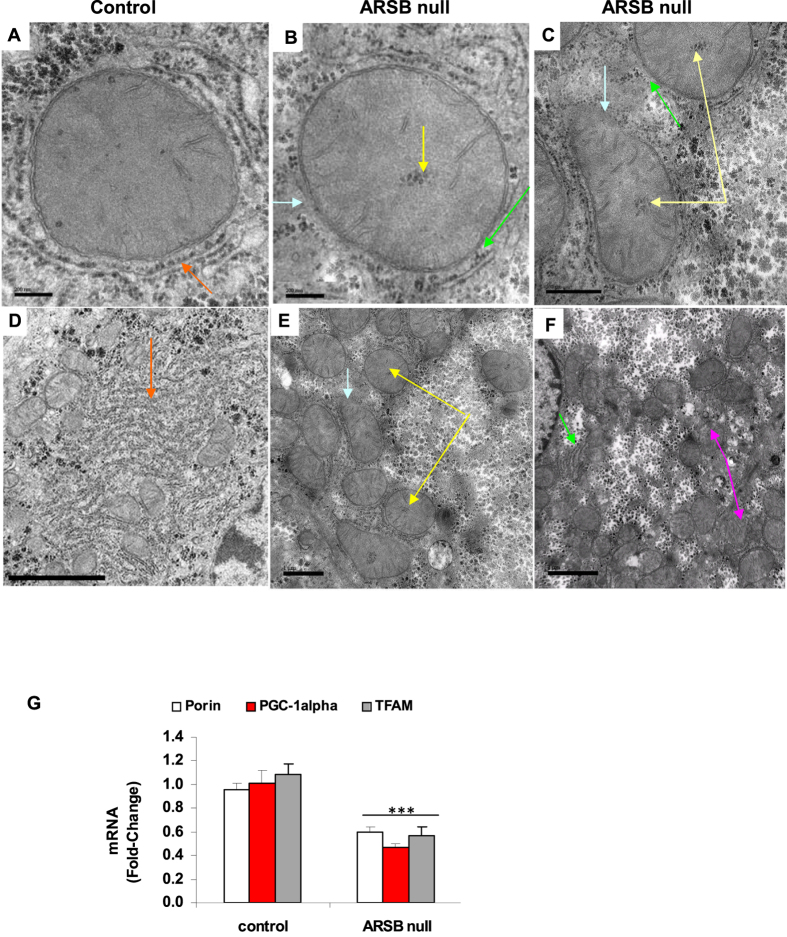
Ultrastructural characteristics and mitochondrial gene expression in hepatic tissue from ARSB-null mice. (**A**) Transmission electron micrographs (EM) of a mitochondrion from the control C57BL/6J mouse liver shows intact mitochondrial membranes and extensive surrounding endoplasmic reticulum (orange arrow) (bar = 200 nm; original magnification = 103,000). (**B**) In contrast, mitochondrion from ARSB-null mouse liver shows unusual central inclusions (yellow arrow), disruption of the surrounding endoplasmic reticulum (ER; green arrow), and discontinuity of the mitochondrial membranes (blue arrow) (bar = 200 nm). (**C**) EM shows marked disruption of the mitochondrial membranes (blue arrow) and prominent central inclusions (yellow arrow), as well as disarray of the surrounding ER (green arrow) (bar = 500 nm). (**D**) EM of control mouse hepatic tissue shows abundant mitochondria and endoplasmic reticulum (orange arrow), without opacification of the surrounding cytoplasm (bar = 2 μm; original magnification = 15,000). (**E**) The ARSB-null mice mitochondria show frequent central inclusions (yellow arrow) and disruption of the mitochondrial membranes (blue arrow) (bar = 1 μm). (**F**) The ARSB-null hepatic cells show extensive opacification of the surrounding cytoplasm, loss of ER (green arrow) and disrupted mitochondria (pink arrow) (bar = 1 μm). (**G**) mRNA levels of three mitochondrial genes were determined by QPCR. The genes porin, TFAM (Transcription Factor A, mitochondrial) and PGC (peroxisome proliferator-activated receptor-gamma coactivator)-1α were significantly reduced in hepatic tissue of ARSB null mice. [AF = ARSB-null female; AM = ARSB-null male; ARSB = arylsulfatase B; CF = control female; CM =  = control male; EM = electron micrograph; ER = endoplasmic reticulum; MMP = mitochondrial membrane potential; TEM = transmission electron microscopy]

Transmission electron micrographs of hepatic tissue from ARSB-null and control mice are presented in Fig. 5(A–F). The images demonstrate frequent disruption of the mitochondrial inner and outer membranes, accumulation of dense particles centrally in the mitochondrial matrix, and marked reduction and disarray of the endoplasmic reticulum in the ARSB null-mouse hepatic tissue (Fig. 5B,C,E,F), compared to age-matched control (Fig. 5A,D). An overall increase in degraded mitochondria is apparent in some of the images of the ARSB-null hepatic tissue (Fig. 5F), compared to the control (Fig. 5D). However, in other regions, the density of the mitochondria appears similar to the control (Fig. 5E).

To further evaluate mitochondrial function, mRNA expression of three mitochondrial genes was determined by QPCR using hepatic tissue from the ARSB-null and control mice (Fig. 5F). Expression of the genes porin, TFAM (transcription factor A, mitochondrial, mtTFA) and PGC (peroxisome proliferator-activated receptor-γ coactivator) -1α was reduced by about 50%, consistent with the observed mitochondrial defects.

### Increases in NADH and NADPH follow decline in ARSB

**Figure 6:**
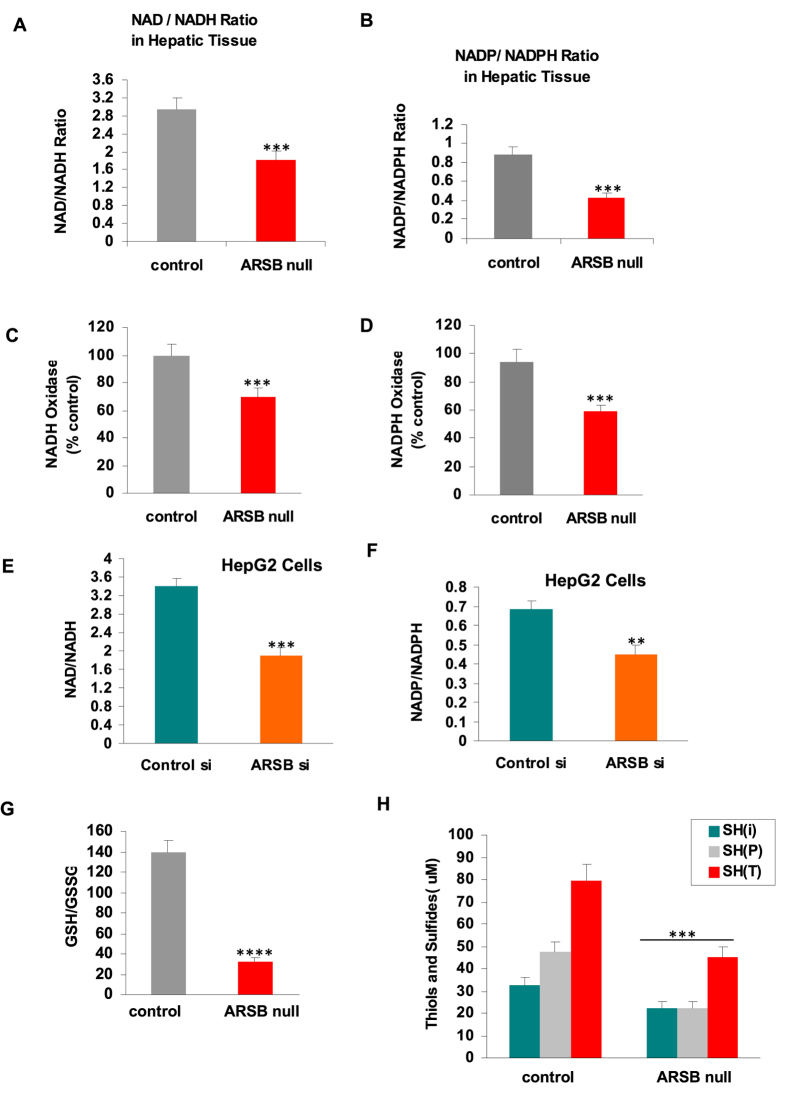
NAD+/NADH and NADP+/NADPH ratios, NADH and NADPH oxidase activity, and GSH/GSSG ratio and thiol content. (**A**) The ratio of NAD+ to NADH is significantly lower in the ARSB-null mouse hepatic tissue, mainly due to increase in NADH, which increased by ~66 ng/mg protein in the ARSB-null mouse hepatic tissue (n = 20). (**B**) In the ARSB-null mouse hepatic tissue, the ratio of NADP+ to NADPH was also less, largely attributable to an increase in NADPH of ~9.5 ng/mg protein (n = 20). (**C**) Consistent with the increase in NADH, the NADH oxidase was significantly less in the ARSB-null mice (n = 12). (**D**) Consistent with the observed increase in NADPH, the NADPH oxidase activity was less in the ARSB-null hepatic tissue (n = 20). (**E**) In the ARSB-silenced HepG2 cells, the ratio of NAD+ to NADH was also significantly less, due predominantly to increase of ~80 ng/mg protein in NADH (n = 3). (**F**) In the ARSB-silenced HepG2 cells, the ratio of NADP+ to NADPH was significantly less, due to increase of ~8 ng/mg protein in NADPH (n = 3). (**G**) In contrast to the increases in the reduced forms NADH and NADPH when ARSB was lower in the ARSB-null mouse hepatic tissue, the ratio of glutathione (GSSG) to reduced glutathione (GSH) was increased, due to decline in reduced glutathione (n = 12). (**H**) Similarly, the level of thiols was significantly reduced in the ARSB-null hepatic tissue, demonstrating an overall decline in reduced sulfur products when ARSB activity was less (n = 12). [ARSB = arylsulfatase B; CF = control female; CM = control male; GSH = glutathione; GSSG = glutathione disulfide].

In the ARSB-deficient mouse hepatic tissue, the ratios of NAD+ to NADH (Fig. 6A) and NADP+ to NADPH (Fig. 6B) were markedly less, compared to levels in the normal controls. The NAD^+^/NADH ratios of control mice were 2.93 ± 0.26 (n = 10). These ratios were significantly lower in the ARSB-null mice (1.80 ± 0.21, p < 0.0001; n = 10). The declines in the ratios were due predominantly to increases in the reduced forms of NADH and NADPH. In the ARSB-null female mouse hepatic tissue, NADH increased to 201.8 ± 12.1 ng/mg protein from 135.8 ± 9.4 ng/mg protein (p < 0.0001), and NADPH increased to 36.5 ± 1.7 ng/mg protein from 26.9 ± 1.2 ng/mg protein in the female mice and to 35.7 ± 1.0 ng/mg protein from 26.9 ± 3.9 ng/mg protein in the male mice (p < 0.0001; p = 0.001). The activities of NADH oxidase (Fig. 6C) and of NADPH oxidase (Fig. 6D) were significantly less in the ARSB-null hepatic tissue (p < 0.0001) than in the control tissue.

Similar changes were demonstrated in ARSB-silenced HepG2 cells. The NAD^+^/NADH ratio decreased to 1.9 ± 0.16 from 3.4 ± 0.18 (p = 0.0004) (Fig. 6E). The NADP^+^/NADPH ratio declined from 0.68 ± 0.04 to 0.45 ± 0.05 (p = 0.003) (Fig. 6F). In the ARSB-silenced HepG2 cells, NADH increased to 229.2 ± 18.6 ng/mg protein from 148.9 ± 11.4 ng/mg protein (p = 0.003) and NADPH increased to 43.6 ± 3.7 ng/mg protein from 35.7 ± 2.6 ng/mg protein (p = 0.04). These findings indicate profound changes in the normal metabolism when ARSB is inhibited.

In contrast to the increases in the reduced forms NADH and NADPH, levels of reduced glutathione and of thiols were less in the ARSB-null mouse liver than in control. The ratio of reduced glutathione (GSH) to oxidized glutathione (GSSG) (Fig. 6G) and the thiol content, including total, inorganic, and protein-associated (Fig. 6H), were significantly less in the ARSB-null hepatic tissue. These declines in the reduced forms of the sulfur-containing compounds contrast with the increases in NADH and NADPH. This difference suggests an overall defect in sulfate reduction when ARSB is diminished, although the overall reducing capacity of NADH and NADPH is increased.

## Discussion

Removal of the 4-sulfate group of N-acetylgalactosamine-4-sulfate at the non-reducing end of chondroitin 4-sulfate (C4S) or dermatan sulfate (DS) is the only known direct effect of ARSB. Reduced availability of sulfate due to decline in ARSB activity may impact on other cellular processes, such as the formation of 3′-phosphoadenosine 5′-phosphosulfate (PAPS) which is required for sulfotransferase reactions. Other processing or utilization of the sulfate that is removed from C4S or DS in mammalian cells has not been elucidated. Sulfate reduction for energy production is recognized in lower organisms, including in protists, algae, and plants^21^,^22^,^23^,^24^,^25^,^26^,^27^,^28^,^29^,^30^. Deficiency of environmental sulfate leads to reduced plant size^22^. Altered mitochondrial structure and function and impaired Complex 1 activity have been reported when sulfate availability is restricted or sulfate transporters are inhibited in plants^21^,^27^,^28^. Decline in mitochondrial membrane potential was found in fibroblasts from children with MPS VI^31^. MPS VI patients characteristically have short stature and multiple skeletal and physiological abnormalities, attributed to accumulation of C4S and DS throughout their tissues.

**Figure 7:**
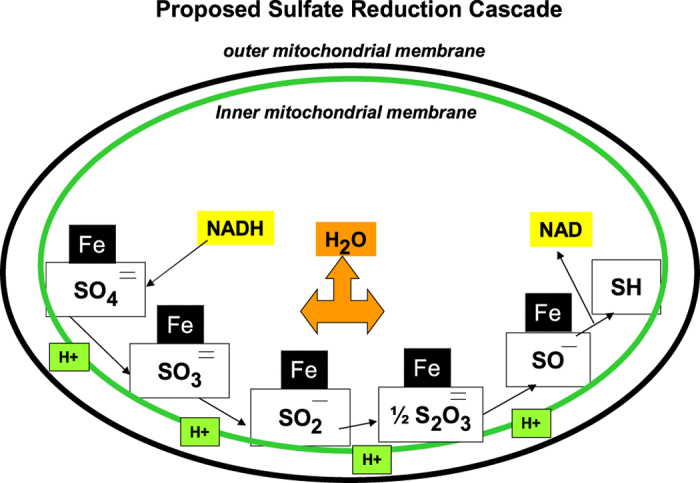
Proposed sulfate reduction pathway in mammalian mitochondria. Cascades of sulfate reduction have been described in plants and bacteria. Mammalian sulfate reduction is hypothesized to involve similar intermediates. Sulfate released from chondroitin 4-sulfate by ARSB might undergo progression reductions from sulfate to sulfite to sulfide, involving iron and NADH.

Although sulfur-containing molecules are abundant and vital in human cells, metabolic effects due to reduced availability of sulfate have not been described previously in mammalian cells. Figure 7 presents a hypothetical model of sulfate reduction in mammalian mitochondria. This schema shows potential intermediates of sulfate reduction. Since there was no sulfotransferase activity in the normal mouse mitochondria (Fig. 1L), we hypothesize that sulfate, either produced in the mitochondrion by ARSB or imported into the mitochondrion, is reduced in a cascade requiring iron and NADH. Normal sulfate reduction can contribute to the formation of organosulfur products, such as glutathione. Also, normal sulfate reduction may contribute to the formation of the Fe-S clusters, and decline in ARSB may impair normal Fe-S cluster formation. Inhibition of formation of normal Fe-S clusters would help to explain the observed decline in Complex 1 NADH dehydrogenase activity, since Fe-S clusters are components of Complex 1.

Decline in available sulfate due to ARSB deficiency may help to explain the reduced oxygen consumption and the enhancement of aerobic glycolysis, the Warburg effect, of malignant cells. Decline in ARSB was reported in human malignancies^12^,^13^,^14^,^15^,^16^, and mechanisms for inhibition of ARSB activity have been identified^9^,^17^,^18^. Acquired deficiency of ARSB activity may arise from impaired oxygen delivery to tissues, since molecular oxygen is required for the post-translational activation of ARSB^9^. Other exposures, including ethanol and high chloride, also can reduce ARSB activity^32^,^33^.

The increase in NADPH when ARSB activity is less may be due in part to impaired sulfotransferase activity, since PAPS (3′-phosphoadenosine 5′-phosphosulfate) utilizes NADPH in sulfate reduction^34^,^35^. Previously, decline in ARSB was shown to reduce sulfotransferase activity and expression of chondroitin 4-sulfotransferase (CHST11)^36^. ARSB-mediated enhancement of glycolytic flux may also contribute to some extent to the increase in the NADH/NADPH levels in the ARSB-null hepatic tissue and the ARSB-silenced HepG2 cells (Fig. 6B,F). No significant changes in expression of glycolytic genes were detected in the cDNA microarray from ARSB-null hepatic tissue, compared to the C57BL/6J control^11^.

We hypothesize that ARSB activity and the associated release of sulfate from C4S or DS are required for normal oxidative metabolism. Also, we hypothesize that sulfate is a functional component of Complex 1 and may be required for normal Fe-S complexation. In the experiments of this report, when ARSB activity declined and sulfate was thereby less available, extracellular acidification rate and lactate production increased, the oxygen consumption rate declined, mitochondrial membrane potential decreased, Complex 1 activity (NADH dehydrogenase) was inhibited, mitochondrial morphology was disrupted, NADPH and NADH increased, reduced glutathione and sulfhydryl (-SH) content were less, and activity of NADH and NADPH oxidases declined. The study findings indicate an association between decline in ARSB activity and enhanced aerobic glycolysis with impaired oxidative metabolism. Findings suggest that ARSB, which requires molecular oxygen for activation, acts as a redox switch which influences and may regulate oxidative metabolism. A mechanism for the declines in NADH and NADPH oxidase activity is unknown at this time. The declines in activity may be related to transcriptional effects due to changes in binding of galectin-3 and SHP2 with C4S when ARSB is inhibited. Shp2 was reported to regulate the phosphorylation of transcription factors HoxA10 and ICSBP, resulting in changes in expression of NADPH oxidase components in myeloid cells^37^. Galectin-3 acts with AP-1 for promoter activation^8^,^9^, and AP-1 has been reported to bind to and activate the NADPH promoter^38^.

The electron micrographs indicate significant effects of decline in ARSB on the structure of the endoplasmic reticulum (ER), as well as of the mitochondria. ER defects may be related to the impact of increased chondroitin 4-sulfation on calcium binding and may contribute to the impaired cellular metabolism.

Endosymbiotic theory supports the bacterial origin of mitochondria, and sulfate reduction occurs in many bacteria. Sulfate-reducing bacteria and archaea metabolize sulfate, reducing it to produce hydrogen sulfide or other sulfur-containing products^29^. Sulfate acts as an electron acceptor in many bacteria, such as *desulfovibrio vulgaris*^30^. The current study findings suggest that mitochondrial sulfate reduction in mammalian cells may have a critical role in the modulation of cell metabolism. Increased attention to the fate of sulfate and the role of sulfatases, sulfotransferases, and glycosaminoglycans in mammalian biochemistry may help to explain fundamental aspects of cellular metabolism and bioenergetics.

## Materials and Methods

### Materials

Eight-week-old, heterozygous ARSB^+/−^ male and female mice were procured from Jackson Laboratories, Bar Harbor, Maine, USA, and housed in the Biological Resource Laboratory at the University of Illinois at Chicago (UIC). All methods were in accordance with relevant guidelines and regulations for animal care. All experimental protocols were approved by the Institutional Animal Care and Use Committee (IACUC) of the UIC and the Jesse Brown VA Medical Center. Mice were fed a standard diet and maintained with routine light–dark cycles. The mice were inbred to develop homozygous ARSB-null mice, and mutation of the ARSB gene was confirmed by genotyping, as previously reported^13^. The homozygous ARSB-null mice were inbred for another two generations for pure lineage. Body weight and length were measured and compared to normal age- and gender- matched C57BL/6J mice. Twelve-week-old control and ARSB-null mice were euthanized (n = 20, divided equally by gender and control vs. null), and livers were sampled. Primary hepatocytes were prepared from the liver tissue and maintained in cell culture, according to an established protocol^39^.

Human HepG2 cells and the primary hepatocytes from ARSB-deficient mice and age- and gender- matched C57BL/6J control mice were used in cell-based experiments. HepG2 cells (ATCC HB-8065; Manassas, VA) were grown under standard conditions, including minimum essential medium (MEM) with 10% FBS, 5% CO_2_, 95% humidity at 37 °C, and with media exchange every 2–3 days. Confluent cells were harvested by EDTA-trypsin, and sub-cultured in multiwell tissue culture plates, as required for the different experiments. Some cell preparations at 60–70% confluency were transfected with ARSB siRNA or by control siRNA for 24 hours, as previously detailed^8^,^11^.

### Isolation of mitochondria

Mitochondria were isolated from mouse hepatic tissue and from cultured hepatic cells using a mitochondrial isolation kit (Abcam, Cambridge, MA), and following the recommended protocol. Appropriate quantities were washed in wash buffer, minced, and placed in the pre-chilled Dounce homogenizer and Isolation Buffer was added. The homogenate was transferred into microtubes with Isolation Buffer, centrifuged at 1000 g for 10 minutes at 4 °C, then the supernatant with Isolation Buffer was centrifuged at 12,000 g for 15 minutes at 4 °C. The pellet was washed and re-suspended in Isolation Buffer with protease inhibitor and centrifugation was repeated. The protein content of the pellet, containing mitochondria, peroxisomes, and lysosomes, was assayed. This pellet, containing a crude mitochondrial preparation, was used in some of the experiments.

To further isolate the mitochondria from lysosomes and peroxisomes, a discontinuous density gradient using OptiPrep^TM^ Density Gradient Medium (Axis-Shield, Sigma) was employed^40^. Gradients in descending concentrations ranging from 30 to 17% were prepared. The diluent buffer consisted of 0.25 M sucrose, 1 mM EDTA, 20 mM Hepes-NaOH at pH 7.4. The prepared tissue extract was mixed with the OptiPrep^TM^ Cell Separation Media to make a final concentration of 10% OptiPrep^TM^. The sample was layered on top of the density gradients, and then the preparation was ultracentrifuged at 145,000 g for 2 h at 4 °C. The lysosome band was located in the top 3 ml of the gradient, removed, and stored on ice. The remaining 4 ml contained the mitochondria and the peroxisomes. This sample was diluted with 3–4 volumes of PBS, transferred into a centrifuge tube, and centrifuged at 18,000 g for 30 minutes at 4 °C. The supernatant was removed, and the pellet was re-dissolved in the diluent buffer. The peroxisome fraction was isolated in the 32% gradient layer and as a loose pellet following repeat ultracentrifugation at 100,000 g for 3 h on a discontinuous gradient ranging from 20–35%. The mitochondrial fraction was isolated in the remaining solution.

### Assays of marker enzymes

Activity assays of the marker enzymes, catalase, acid phosphatase, and succinate dehydrogenase, were performed to confirm the effectiveness of separation of peroxisomes, lysosomes, and mitochondria. (Table 1) Assays were performed using established procedures^41^.

The peroxisome marker catalase was quantified by the catalase enzyme assay (Sigma) which proceeded by combining 100 μl of the preparation of peroxisomes, lysosomes, mitochondria, or the crude mitochondrial preparation to a quartz cuvette with 400 μl of assay buffer. The preparation was mixed by inversion. The reaction was started by adding 0.5 ml of UV Assay Substrate Solution (20 mM hydrogen peroxide) and mixed by inversion. Decline in the absorbance signal at 240 nm was followed for 1 min and activity was expressed as units. One unit of catalase will decompose 1.0 micromole of hydrogen peroxide to oxygen and water per minute at pH 7.0 and 25 °C at a substrate concentration of 10 mM hydrogen peroxide.

Succinate dehydrogenase activity measures the conversion of the substrate succinate to fumarate using nitroblue tetrazolium (NBT; Calbiochem). NBT is the artificial electron acceptor which changes to purple as succinate is modified^41^. NBT (0.1 mg of 2.5 mg/ml), 0.1 ml of 1%Triton, 0.1 ml of 100 mM Na succinate, 0.2 ml of 200 mM Na-Phosphate buffer (pH 7.4), and 0.5 ml of each of the cell fractions were incubated at 37 °C for 30 minutes. The reaction was stopped by 2.0 ml of 2% sodium dodecyl sulfate, and absorbance was read at 630 nm in a spectrophotometer. OD results were compared among the samples using the control value of 1.0 for crude mitochondria from the hepatic tissue of the normal control mice.

Acid phosphatase activity was used as a marker of lysosomes in the organelle preparations. Acid phosphatase cleaves terminal phosphate groups, and activity is maximal at acid pH. The colorless compound para-nitrophenol phosphate (pNPP) was used as the substrate, since when phosphate is cleaved, the resulting para-nitrophenol is yellow and can be detected at ~405 nm in a spectrophotometer. For the assay, 0.1 ml of buffer (0.25 M glycine-HCl, pH 3.0 with 0.5% Triton X-100} and 50 μl of 50 mM pNPP in water were combined with 0.1 ml of a 1:10 dilution of the cell fraction of interest^41^. Tubes were incubated for 30 minutes at 37 °C. The reaction was stopped by addition of 2.75 ml of ice-cold 0.2 M sodium phosphate (pH 12) to each tube. Absorbance was read at ~405 nm in a spectrophotometer. OD results were compared among the samples, using the control value of 1.0 for crude mitochondria in the hepatic tissue of the normal control mice.

### Arylsulfatase B activity, sulfotransferase activity, total sulfated glycosaminoglycans, and chondroitin 4-sulfate assays

Determinations of ARSB activity were performed using a fluorimetric assay with the substrate 4-methylumbelliferyl sulfate (4-MUS)^15^,^16^. 20 ml of cell or tissue homogenate and 80 ml of assay buffer (0.05M Na acetate buffer, pH 5.6) were combined with 100 ml of substrate (5 mM 4-MUS in assay buffer) in wells of a microplate. The microplate was incubated for 30 minutes at 37 °C. The reaction was stopped by 150 ml of stop buffer (Glycine-Carbonate buffer, pH 10.7), and fluorescence was measured at 360 nm (excitation) and 465 nm (emission) in a microplate reader (FLUOstar, BMG LABTECH, Inc., Cary, NC).

Sulfotransferase activity was determined using the Universal Sulfotransferase Activity kit (R&D, Minneapolis, MN), which assesses the sulfotransferase activity of all the sulfotransferases in the cells or tissue tested^36^. 3′-Phosphoadenosine-5′-phosphosulfate (PAPS) is the sulfate donor for the sulfotransferase reaction. PAPS (10 μl, 1 mM), acceptor substrate (10 μl), and coupling phosphatase (3.5 μl, 100 ng/μl) were combined with 25 μl/well of the cell or tissue suspension in which overall sulfotransferase activity was to be determined in the wells of a microtiter plate. For negative controls, assay buffer was substituted for the cell suspension. The mixture was incubated for 20 min at 37 °C, then 30 μl of malachite green was added and the mixture gently tapped. De-ionized water (100 μl) was added to each well, and color was developed. Optical density of each well was read and compared among the different wells. The amount of product formation was calculated using a phosphate standard curve, following subtraction of the reading of the negative control. The 3-inorganic phosphate released by the coupling phosphatase and detected by malachite green phosphate detection reagent, was proportional to the 3′-phosphoadenosine-5′-phosphate generated, thereby indicating the extent of the sulfotransferase reaction which utilized the sulfate group of PAPS.

Measurements of total sulfated glycosaminoglycans (GAGs) and chondroitin 4-sulfate (C4S) were performed as previously described^16^,^32^. Briefly, the Blyscan^TM^ assay kit (Biocolor Ltd, Newtownabbey, N. Ireland) was used for detection of the sulfated GAG, based on the reaction of 1,9-dimethylmethylene blue with the sulfated oligosaccharides in the GAG chains. C4S content was determined following immunoprecipitation with C4S antibody (LY111; Amsbio, Lake Forest, CA). For measurement of mitochondrial chondroitin 4-sulfate, the mitochondrial fraction was prepared from mouse hepatic tissue by differential centrifugation, and chondroitin 4-sulfate concentration was determined by the Blyscan assay following immunoprecipitation with chondroitin 4-sulfate antibody, as described above.

### Mitochondrial membrane potential

Mitochondrial membrane potential was determined by a commercial kit (Abcam) in which change in color of the cationic, lipophilic dye JC-10 is used to detect changes in the membrane potential. In normal cells, JC-10 concentrates in the mitochondrial matrix where it forms orange fluorescent aggregates (emission 590 nm). However, in apoptotic and necrotic mitochondria, JC-10 diffuses out of the mitochondria, and changes to monomeric forms and shows green fluorescence (emission 520 nm). Hence, when membrane potential increases, the 590/520 nm ratio increases; in contrast, when membrane potential decreases, the 590/520 nm ratio is reduced. J-10 was used to measure the mitochondrial membrane potential (MMP), noting that the monomeric dye is green, whereas the aggregate associated with increased MMP is orange, and the ratio of orange to green absorbance characterizes the MMP. Studies were performed using the mitochondria-containing fraction of the hepatic cells and tissue. The fluorescent intensities for both JC-10 aggregates in the mitochondrial matrix and monomeric JC-10, which has diffused out of the mitochondria, were measured at Ex/Em = 490/520 nm and 540/590 nm, respectively, with a microplate reader (FLUOstar), and the ratios of emission at 590/520 were compared among the different samples.

### Transmission electron microscopy of hepatic mitochondria

Hepatic tissue was obtained from the ARSB-null mice and age- and gender matched controls, promptly minced into 1 mm^3^ pieces, and placed in cold 2.5% glutaraldehyde in 0.1 M cacodylate buffer. Tissue was subsequently trimmed and placed into fresh 3% glutaraldehyde in 0.1 M cacodylate buffer and stored at 4 °C until further processing. Post-fixation, the tissues were treated with 1% osmium tetroxide and dehydrated in a series of ethanols up to 100% and then transitioned to 1:1 propylene oxide solvent and further processed by infiltration of epoxy resin 1:1 and 3:1 pure resin (Lx112 H resin) on a rotor overnight. Tissue was embedded in Lx112 epoxy resin and cured in a 60 °C oven. One micron sections were placed on glass slides and stained with toluidine blue stain, and ultrathin 90 nm sections were placed on 200 mesh copper standard square grids. Two sample blocks were cut per sample, and grids were stained with 2% uranyl acetate aqueous and lead citrate. Samples were viewed at different magnifications in the JEM MEOL 1220 transmission electron microscope in the Research Resource Center of UIC. Digital images were obtained using the Erlangshen ESW 1000W 785 camera and downloaded.

### Measurements of NAD+, NADP, NADP+, and NADPH

Nicotinamide adenine dinucleotide (NAD) and nicotinamide adenine dinucleotide phosphate are crucial coenzymes in metabolic oxidation-reduction reactions and contain both an adenosine and a nicotinamide nucleotide, coupled by a pyrophosphate link. Measurements were performed in human HepG2 cells and mouse hepatic tissue using established assays (Abcam), and values are expressed as the ratio of NAD+ to NADH and NADP+ to NADPH following ARSB or control silencing and in the ARSB-deficient or control mice. Total NAD and NADH in the samples was measured spectrophotometrically by using a specific developer, and then NADH in the samples was detected following decomposition of the NAD+ in the mixture by heating at 60 °C for 30 minutes. Optical density was determined at 450 nm, and NADH concentrations were compared with a standardized calibration curve. NAD was calculated as the difference between total NAD and NADH. NAD^+^/NADH ratio was calculated and analyzed. The assay was specific for NAD/NADH, and did not detect NADP/NADPH.

Total NADP^+^ and NADPH and NADPH were measured in the hepatic cell and tissue samples by a commercial assay (Abcam). Total NADP (NADP+ and NADPH) was detected spectrophotometrically, and then the NADP+ was decomposed, yielding NADPH which was detected by measurements of optical density at 450 nm, and quantified using a standardized calibration curve. This assay was specific for NADP/NADPH and did not detect NAD/NADH.

### NADH and NADPH oxidase activity

NADH and NADPH oxidase activities were determined by assays that measure the products of the reactions. The values were compared between control, control si, and ARSB si using calibrated standards. NADH oxidase was determined using a standardized, spectrophotometric assay^42^. The reagents (Sigma-Aldrich, St. Louis, MO) 250 mM potassium phosphate buffer pH 7.0 at 30 °C, 1 mM flavin adenine dinucleotide (FAD) solution, and 2.0 mM β-NADH were combined with deionized water in a 3 ml cuvette. The tissue sample (0.10 ml) in an enzyme diluent composed of 30 mM potassium phosphate buffer with 0.1% (w/v) BSA pH 7.5 at 30 °C was mixed with the FAD-NADH-buffer and spectrophotometric readings at OD 340 were recorded for five minutes. Units were quantified using the value of 6.22 as the millimolar extinction coefficient of β-NADH at 340 nm. One unit of NADH oxidase was defined as the amount of enzyme required to oxidize 1.0 umole of NADH in one minute.

NADPH oxidase activity was quantified by a microplate fluorometric assay^43^. The hepatic tissue membrane fraction (~10 μg protein) was incubated with dihydroethidium (10 μM) in PBS/DTPA with NADPH (50 μM) and calf thymus DNA (1.25 ug/ml) in a volume of 120 ul for 30 minutes at 37 °C in the dark. Total fluorescence was followed in the microplate reader.

### Measurement of serum lactate

Serum lactate was measured in the ARSB-deficient and control mice under non-stress conditions. L(+)-Lactate concentration was determined by an enzymatic assay (Sigma-Aldrich) in which a lactase enzyme produced a colorimetric (570 nm) product that was proportional to the lactate present in the serum samples.

### Oxygen consumption rate and extracellular acidification rate

These measurements were performed using fluorescent indicators in HepG2 cells following ARSB- or control- silencing for 24 h. Control and ARSB-silenced HepG2 cells were treated with the electron transport chain (ETC) inhibitors Antimycin A (AMA; 1 μM) and Rotenone (1 μM), or with carbonyl cyanide-p-trifluoromethoxyphenylhydrazone (FCCP; 3.3 μM), an uncoupler of oxidative phosphorylation. The effects of these inhibitors were compared between the ARSB-silenced and control-silenced cells. Cells were exposed to the inhibitors at time 0 and maintained at 37 °C with 5% CO_2_ for the duration of the experiment. Readings were taken every 7 minutes for 2 hours from wells with no added test substrate or from wells into which only a single test substrate had been inoculated. Differences between ARSB-silenced and control-silenced readings were calculated per mg protein using measurements of the total cell protein per well. Readings were presented graphically at each time point for each condition. Statistical significance of the differences was calculated by unpaired t-tests, two-tailed, using the slope of the linear portion of the curve obtained over the first 60 min, and using the mean value of the determinations which were performed in six distinct biological samples for each of the eight different test preparations.

Oxygen consumption rate (OCR) was measured in the control and treated HepG2 cells using the MitoXpress^®^ Xtra kit (Luxcel). In this assay, MitoXpress^®^-Xtra, a water-soluble oxygen-sensitive phosphorescent probe, is quenched by O_2_ through molecular collision. Thus, the amount of fluorescence signal is inversely proportional to the amount of extracellular O_2_ in the sample. Rates of oxygen consumption are calculated from the changes in fluorescence signal over time. The fluorescence was measured in a fluorescence plate reader (FLUOstar), with excitation and emission wavelengths of 365 nm and 650 nm, respectively.

Extracellular acidification rate (ECAR) in the control and ARSB-silenced HepG2 cells was determined using the pH-Xtra kit (Luxcel). pH-Xtra is a soluble fluorescent probe, and its emission is modulated by the pH of the solution. Changes in intensity indicate changes in extracellular pH, with decreasing pH (acidification) causing an increase in probe emission. The fluorescence was measured in a fluorescence plate reader (FLUOstar) with excitation and emission wavelengths of 380 nm and 615 nm, respectively. Cells were untreated or treated with inhibitors, as described above.

### Reduced glutathione and glutathione (GSH/GSSG) assay

Reduced glutathione (GSH), GSSG (glutathione disulfide), and total glutathione (GSH+ GSSG) in the control and null mouse hepatic tissue were determined (Glutathione Detection Kit, BioVision, Inc., Mountain View, CA)^9^. Samples were collected in 6N perchloric acid on ice to avoid oxidation of labile GSH. GSH was determined by fluorescence of the reaction of cell samples with O-phthalaldehyde (OPA), which does not react with GSSG. GSH-GSSG total was determined by first adding a reducing agent to convert GSSG to GSH, and then reacting with OPA to produce fluorescence. To measure GSSG specifically, a GSH quencher was added initially to remove GSH, preventing the reaction with OPA, but not interacting with GSSG. Reducing agent was then added, and destroyed excess quencher and converted GSSG to GSH. Fluorescence was detected in a fluorescence plate reader (FLUOstar) with excitation and emission wavelengths of 340 nm and 420 nm, respectively.

### Total, inorganic, and protein sulfhydryl determinations

Sulfhydryl (thiol) content in the control and ARSB-null hepatic tissue was determined by a commercial assay (Molecular Probes, Eugene, OR)^9^. Inorganic thiols in the samples reduced a disulfide bond, thereby releasing the active enzyme papain from its inactive S-S form. The activity of the enzyme was detected using the chromogenic papain substrate, N-benzoyl-L-arginine, *p*-nitroanilide (L-BAPNA). The intensity of the developed color was proportional to the thiol quantity in each sample. To measure the total (inorganic and protein-associated) thiols, cystamine was added to the reaction, enabling the detection of poorly accessible thiols on proteins with high pKa values. The disulfide cystamine has an exchange reaction with protein thiols, yielding 2-mercaptoethylamine (cysteamine), which then releases active papain. Active papain then acts on L-BAPNA to develop color; absorbance is measured in a spectrophotometer at 410 nm. The protein sulfhydryl component is calculated as the difference between total and inorganic components.

### Complex 1 activity by NADH dehydrogenase assay

Mitochondrial Complex 1 activity was detected using a colorimetric microplate assay in which change in the NAD+ production was measured over time (Abcam) in the mitochondria from HepG2 cells following ARSB- or control- silencing and from mouse hepatic tissue of ARSB-null and control mice. Complex 1 capture antibodies were pre-coated on the microplate wells. Samples were added and the Complex 1 target was immobilized. Activity was determined by the oxidation of NADH to NAD+ and the synchronous reduction of a dye leading to increased absorbance at 450 OD. This assay detects the diaphorase-type activity of Complex 1 (oxidation of reduced form of NADH; NADH dehydrogenase activity) and is not dependent on ubiquinone.

### QPCR of mouse mitochondrial genes

QPCR was performed using established techniques to determine the mRNA expression of three mouse mitochondrial genes. mRNA expression was determined: for TFAM (Transcription Factor A, mitochondrial; NM_009360) (left primer) AGCAGGCACTACAGCGATACA and (right primer) TCTACCTTTCCCATTCCCTTC; for porin (NM_011691) (left primer) GAAGTGAACACAGACAACACC and (right primer) CAACCCTCATAGCCAAGCAC; and for PGC (peroxisome proliferator-activated receptor-gamma coactivator)-1α; NM_008904.2) (left primer) AAACAGGAACAGCAGCAGAGA and (right primer) TGGGGTCAGAGGAAGAGATAAA. Transcript abundance was compared between the hepatic tissue of the ARSB null mice and the age-matched C57BL/6J control mice. Primers were selected using Primer 3 44.

### Statistics

Data are presented as mean ± standard deviation (S.D.) of at least three biologically independent measurements with technical replicates of each measurement, unless stated otherwise. Statistical analysis was performed using GraphPad InStat Software (GraphPad Software, San Diego, CA), and was determined by unpaired t-tests, two-tailed, unless stated otherwise. A p-value of ≤0.05 was considered significant, and ****represents p ≤ 0.0001; ***represents 0.0001 < p ≤ 0.001, ** represents 0.001 < p ≤ 0.01 and *represents 0.01 < p ≤ 0.05.

## Additional Information

**How to cite this article**: Bhattacharyya, S. *et al*. Restriction of Aerobic Metabolism by Acquired or Innate Arylsulfatase B Deficiency: A New Approach to the Warburg Effect. *Sci. Rep.* **6**, 32885; doi: 10.1038/srep32885 (2016).

## Figures and Tables

**Table 1:** Marker enzyme activity following organelle isolation.

Enzyme Activity	Arylsulfatase B Null Hepatic Tissue				Control Hepatic Tissue			
	Crude Mitochondrial Preparation	Lysosomes	Mitochondria	Peroxisomes	Crude Mitochondrial Preparation	Lysosomes	Mitochondria	Peroxisomes
Acid Phosphatase (fold-change)	0.76 (0.02)	1.63 (0.02)	0.06 (0.001)	0.006 (0.002)	1.00 (0.03)	2.05 (0.07)	0.10 (0.004)	0.005 (0.003)
Catalase (Units)	528 (24)	0 (0)	138 (19)	1526 (66)	485 (17)	0 (0)	129 (17)	1509 (56)
Succinate Dehydrogenase (fold-change)	0.76 (0.08)	0.09 (0.008)	1.50 (0.07)	0.073 (0.008)	1.00 (0.063)	0.10 (0.001)	2.11 (0.10)	0.082 (0.002)

S.D. = standard deviation.*One unit of catalase will decompoase 1.0 micromole of hydrogen peroxide to oxygen and water per minute at pH 7.0 and 25 °C at a substrate concentration of 10 mM hydrogen peroxide.
